# Machines like us scientists? AI tools for mining the scientific literature in basic biomedical science

**DOI:** 10.1038/s44319-025-00522-5

**Published:** 2025-07-10

**Authors:** Blanche Schwappach

**Affiliations:** Dean’s Office, University Medical Center Hamburg-Eppendorf, Martinistraße 52, 20246 Hamburg, Germany

**Keywords:** Careers, History & Philosophy of Science, Methods & Resources

## Abstract

Like any new methodology, literature analysis by Large Language Models (LLMs) requires extensive tests by experienced scientists and discussion within specific fields such as cell biology.

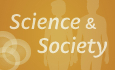

The rapid progress in AI research and its applications herald changes to each step of the scientific process that creates new knowledge: literature analysis, hypothesis generation, experimental design including controls, primary data processing and analysis, figure and table production, manuscript writing, peer review and publication. The individual steps involve different human capabilities such as summarization, multimodal analysis, understanding, reasoning and articulation in natural language. Benchmarking how humans and machines fare in comparison is the subject of whole research fields. In this context, literature analysis by Large Language Models (LLMs) seems a mature and confined topic, which I aim to address here from the perspective of a molecular cell biologist, albeit not in a systematic way.

“The rapid progress in AI research and its applications herald changes to each step of the scientific process that creates new knowledge: literature analysis, hypothesis generation, experimental design including controls, primary data processing and analysis, figure and table production, manuscript writing, peer review and publication.”

“All three tested tools provide a very good summary of the debate on protein transport in the Golgi.”

“It’s about machines like me and people like you and our future together… It will happen. With improvements over time…we’ll surpass you…and outlast you…even as we love you,” says the robot Adam in Ian McEwan’s visionary novel *Machines like me*. Published in 2019, the novel bore no specific message to scientists other than having to realize that science is yet another human activity in which machines could surpass us. Six years later, the question that emerges is indeed, “Which aspects of being a scientist can be supported or replaced by machine tools?.”

## The scholarly craft of literature analysis—past and present

Until the end of the last millennium, it was possible to stay on top of one’s field by following a few general and dedicated journals in combination with regular attendance to conferences. Literature analysis went way beyond reading the abstract and involved critical assessment of the research problem, the methods, the primary data where available, and the conclusions of a paper. With the explosion of the number of scientists in biomedical research and the number of pertinent journals, a comprehensive, deep immersion in the latest developments has become impossible even for senior researchers. In the area of Science, Technology and Medicine, the number of annually published papers are estimated to be around three million. Moreover, as the practice of sharing data in reliable outputs such as preprints, unstructured or structured databases is gaining momentum, the relevant publicly available items are likely to surpass this number by far.

The resulting embarrassment of riches has given extreme weight to the abstract of the article as the main and often only information being used by the respective community. Given this current overwhelming situation and ensuing limitations, the scholarly craft of reading the literature and thereby analyzing hypotheses, data and conclusions, is ideally suited to benefit from the use of LLMs. Analyzing the state of the art, formulating open questions, and generating a testable hypothesis are closely linked steps. Hence, the performance of LLMs in generating hypotheses is a highly intriguing topic.

“…the scholarly craft of reading the literature and thereby analyzing hypotheses, data and conclusions, is ideally suited to benefit from the use of LLMs.”

LLM-based tools as a breathtakingly faster, more thorough and comprehensive option for mining the literature might effectively support managing the huge number of scientific publications. Will it be able to achieve the general human quality of digesting the literature? Intelligently applied, LLMs might enable humans to produce better summaries of a field and offer an improved delineation of the open questions. Time to query some of the available tools against current expert knowledge.

## Current test cases from membrane biology

Here, I present 3 practical test cases from the domain of molecular cell biology to start an informed discussion. First, we are taking the perspective of a whole field on the function of a central organelle within the secretory pathway of the cell, the Golgi apparatus. Second, we will look at one individual protein in the context of a pathway with a dedicated function exerted by many protein complexes together. Third, we will ask a simple question about a single protein’s function in the absence of sufficient primary data directly addressing this problem. These examples reflect three different levels of resolution: a big-picture debate of a whole field, a pathway studied as part of a field, and an individual protein’s function. I chose these test cases in areas that I know enough about to be able to judge the merit of the answers.

I employed tools specifically harnessing LLMs for science, such as ChatGPT o3-mini, SciSpace or Scopus AI. To avoid some of the issues related to the limitations of the LLMs’ training set—lack of specific information domains or updated information—the latter two use retrieval-augmented generation (RAG) technology. Hence, in addition to using ChatGPT—most likely o4—they access constantly updated specific data sets, such as pdf files of scientific articles (SciSpace) or their abstracts (Scopus AI), to benchmark and improve their answers. The test occurred over the course of a year and resulted in a glimpse into the astonishing, ultrafast evolution of these tools.

## Test case 1: the debate about transport mechanisms in the Golgi apparatus

This topic reflects ca. 50 years of research. It is an ideal example to illustrate how science works in an epistemological sense. Mechanistic hypotheses based on observations obtained by specific techniques and data sets culminated in the first model – vesicular transport – of how secretory proteins pass through the Golgi. Briefly, it states that cargo molecules move between different parts of the Golgi by way of vesicles that bud from and fuse with different parts of the organelle. Results obtained by new techniques suggested an alternative model: cisternal maturation. The key tenet of this model is that parts of the organelle, the cisternae, change identity with the cargo molecules maturing towards release from the Golgi. Tension between these competing models did not cause a full paradigm shift but rather resulted in a more differentiated and more integrated view of the different sub-processes and mechanisms. The debate has stimulated further research in the field that has since addressed different cargo molecules in diverse model organisms and cell types and uses a multitude of methods. Over time, scientists proposed several additional models that complemented or integrated the two foundational models.

The textbook section presented in Bruce Alberts’ *Molecular Biology of the Cell* still provides the gold standard of a succinct and accessible summary of transport mechanisms in the Golgi apparatus. It clearly describes the two models of vesicular transport and cisternal maturation, indicates their probable coexistence as well as their physiological relevance. The text avoids potentially confusing details such as the historical development of the debate or differences in Golgi structure in different cell types. Similarly, a combination of a few high-quality reviews can well orient a reader with basic knowledge in molecular cell biology within the debate (Arab et al, 2024; Glick and Luini, 2011; Mironov and Beznoussenko, 2019) and link to current research. In conclusion, at this stage, the “classical” ways of understanding the topic still work very well. The textbook provides a high-quality overview in a short time period. The reviews offer significant historical and technical depth and give access to cutting-edge developments.

How do ChatGPT o3-mini, SciSpace or Scopus AI compare on this query? Systematic documentation of the prompts used and the ensuing interaction with the LLMs are beyond the scope of this commentary but some examples for Test case 1 are in Box 1.

All three tools provide a very good summary of the debate. The epistemological angle of the query as reflected in the word “debate” or even just “models” resulted in information on how the field obtained and conceptualized the summarized insights. The LLM-based answers proved to be sensibly structured and pointed to limitations of each model. They contained suggestions of approaches and further experiments to improve our understanding of protein transport in the Golgi. Hence, someone versed in the basics of the field can definitely have a stimulating interaction with any of the tools. I will discuss their comparative advantages comprehensively for all three queries below.

Would the use of the tools or any one of them be superior to the textbook? At this stage, no, because the structure and concise access provided by the textbook still stand out, including its figures. Do the LLM-based tools proffer better ideas for further research than the review articles? Not yet, as the suggestions remain very generic and quickly change focus as the discussion deepens. However, as Penadés et al (2025) have discussed, this depends on the tool used and on the prompt provided – in other words, the situation could, most likely will, change soon. In addition, high-quality review articles take many months to write and for many fields, in particular interdisciplinary, emerging fields, they do not exist. This opens a market for the LLM-based tools even if high-profile review articles currently surpass them in quality. As in many other areas, updates produced by LLMs may integrate into dynamic versions of textbooks or review articles originally written by human authors.

Box 1 The queries started with simple prompts such as “What models characterize the debate about how transport in the Golgi works?” or “How and when did the debate about transport in the Golgi apparatus arise?”. The two tools directly geared for scientific literature mining (SciSpace and Scopus AI) immediately respond with rephrased questions to correct grammar and enhance precision, i.e., “What are the models that characterize the debate about transport in the Golgi apparatus?” (Scopus AI) or “What are the different models that have been proposed to explain the mechanisms of transport in the Golgi apparatus?” (SciSpace, Deep Review mode). They give an indication of how they process the query—Scopus AI by adding a keyword search to the query (“(“Golgi apparatus” OR “Golgi body” OR “Golgi complex” OR “Golgi”) AND (“transport” OR “trafficking” OR “movement” OR “distribution”) AND (“model” OR “simulation” OR “framework” OR “theory”) AND (“vesicle” OR “membrane” OR “protein“OR “cargo”) AND (“cell” OR “cellular” OR “cytoplasm” OR “organelle”)”) and SciSpace by requesting clarifications (“Could you please clarify or narrow your search query regarding the models explaining the mechanisms of transport in the Golgi apparatus?”) and then suggesting clarifications such as “Specific Focus”, “Purpose of the Research”, “Contextual Relevance” or “Depth of Detail”.

All tools are good at organizing the information with short introductory summaries, structured content and tabular (SciSpace, Scopus AI) or mind-map-like (Scopus AI) representations as well as concluding summaries. The two tools directly geared for scientific literature mining provide direct links to the pertinent pdf files (if accessible) and also suggest follow-up searches as “Related Questions” (SciSpace: “In what ways do the cisternal maturation and vesicular transport models complement each other in explaining Golgi transport?” or “How do small GTPases regulate vesicle formation and cargo sorting during Golgi transport?”) or “Go deeper and Emerging Themes” (Scopus AI: “Endosome-to-Golgi Trafficking” or “Golgi in Cancer Therapy”).

## Test case 2: “Is Get3 a chaperone?”

Chaperones are molecules that aid or protect the correct folding state of proteins. Yeast Get3 or animal TRC40 are names of a highly conserved ATPase that plays a central role in a protein-targeting pathway to the endoplasmic reticulum (ER). Most of the original articles strictly consider Get3 a “targeting factor” although they acknowledge chaperone-like aspects of its function (Favaloro et al, 2008; Stefanovic and Hegde, 2007). However, the tight association of *GET3*-related genes with resistance to general stresses and the evolutionary occurrence of Get3-related proteins independent of other components of the targeting pathway argues that a chaperone might be amongst their original functions. In fact, some current work suggests that Get3 is a dual-function protein (Voth et al, 2014; Powis et al, 2013). Hence, in the current literature, the word “chaperone” carries two different meanings for this protein: a targeting factor that uses its capacity to bind hydrophobic regions of a protein and its ATPase activity for capture-release cycles of the client precursor protein; and general chaperone. The two interpretations of the Get3 chaperone lead to different research paths. Hence, the query “Is Get3 a chaperone?” is typical of someone with foundational training in molecular cell biology starting a specific project based on the state of the field.

The textbook uses the term “chaperone complex” for a complex of Get3 with other factors involved in the capture of the client without further differentiation. The text emphasizes common principles of targeting factors. This summary provides no impulse to further investigate the chaperone function of Get3. As opposed to the literature on transport in the Golgi, there is no debate about different interpretations of Get3 function. Hence, a graduate student entering this field would have to perform a sophisticated survey and synthesis of the primary literature to detect and spell out the two characterizations of Get3. This appears as a warranting task for LLM.

Indeed, all tools live up to the expectation and clearly delineate all evidence present in the literature for the dual functionality of Get3. Minimal prompts lead to usefully structured summaries and, most importantly, pinpoint to the relevant publications. However, ChatGPT o3-mini^ref^ invented a reference with a completely plausible title. When asked about this reference, the answer was: “I apologize for the confusion. The reference for Metz W et al (2006) was intended as an illustrative example synthesizing multiple reports on Get3’s involvement in stress responses, rather than a direct citation from a single, identifiable paper. In compiling a summary of the literature on Get3’s dual roles, some details can sometimes merge findings from different studies.” This response illustrates the substantial risk of using ChatGPT without prior knowledge of the field and emphasizes the value of RAG using direct links to original pdf files as provided by SciSpace and Scopus AI.

## Test case 3: what is the transport substrate of the transporter SLC33A1?

This is the test case where my personal analysis suggests that the state of the art reflected in the literature lacks sufficient support by direct evidence. Acetyl-CoA is the accepted cognate transport substrate of SLC33A1, without a sufficient number of key experiments to support this. For a transporter, the gold standard of assigning its substrate is a direct transport assay, ideally both in vitro, using reconstituted protein, and in vivo, using a cellular system, one lacking the transporter and one over-expressing it. As far as SLC33A1 is concerned, there is no in vitro experiment in the literature, presumably because the protein is difficult to purify (Csala et al, 2007). There is one experiment demonstrating increased uptake of radioactive Acetyl-CoA by semi-permeabilized HeLa cells transfected with a control plasmid or one driving the expression of SLC33A1 (Kanamori et al, 1997). Since, the field has described many cellular and organismic phenotypes of SLC33A1 knockout or over-expression without ever repeating or further corroborating the basic function as an Acetyl-CoA transporter.

Here we have left textbook knowledge and the LLM-based tools might offer the possibility to uncover additional evidence, for example, from publications not directly studying SLC33A1, or may even help to generate alternative hypotheses. However, this is where the awesome power of the tools tested here stops, at least for the time being. Due to the large number of experiments building on the original work (Kanamori et al, 1997), many abstracts repeat the assignment of Acetyl-CoA as a transport substrate without adding additional experiments directly testing transport activity. Furthermore, there are several review articles focusing on SLC33A1 (Hirabayashi et al, 2004; Hirabayashi et al, 2013) as well as papers from the field of human genetics that present mutations linked to disease (Lin et al, 2008). None of these papers provides functional experiments directly addressing transport. Yet, their titles often refer to this function, although the primary evidence is far from the gold standard. Given this bias, the tools were unable to suggest an alternative substrate or bioinformatic strategies for generating hypothetical ones. The summaries do not critically challenge the literature and, at this stage, require critical human thought.

As of March 2025, this still held true when probed with more suggestive prompts such as “Give the original reference with the original data showing that SCL33A1transport Acetyl-CoA - no review article.” or “Can you present alternate hypotheses for what the substrate of SLC33A1 could be if it is not Acetyl-CoA?” In April, Zhou et al (2025) provided data from purified human SLC33A1, which corroborates the assignment of Acetyl-CoA as the cognate substrate. This work will enable further exploration of the links between the biophysical features of SLC33A1 transport activity and the phenotypes linked to SLC33A1 dysfunction.

## An exemplary selection of tools

Here are some general comments on the three tools used: First, SciSpace links to the largest number of references. It provides open-access articles directly within the tool, offers combinatorial options for sorting and extracting key information, and enables interaction with the article in a plethora of formats: podcast, chat with the pdf, extraction of experimental parameters and so on. It seamlessly embeds the query and the resulting literature digest in workflows for text production. This depth can be useful to an expert in the topic, but also poses a risk when used by less experienced scientists.

Second, the reasoning model ChatGPT o3-mini as opposed to earlier attempts with ChatGPT4 delineates the debate with elegance and reasonable judgment on scientific relevance. Yet, some small proportion of the rendered content was invented, which clearly represents a problem for any reader to whom the topic is new.

Third, as Scopus AI draws on the content of the Scopus database, it is custom-made for scientific use and institutional provision. Answers to queries rely on abstracts and bibliographic metadata starting from 2003. The basic idea is to limit the material to safe, “vetted” publication abstracts with retracted publications removed. This clearly overemphasizes the impact of the abstract and falls short of generating a critical assessment of the original data. Because it works with tens of millions of original full-text articles and preprints, SciSpace gets much more out of the existing literature—at a higher risk of pulling up irrelevant references and including non-reviewed information. Importantly, none of the tools was able to make a clean distinction between the information retrieved from review articles and original research articles, respectively. This shortcoming represents a substantial barrier to digging deeper into evidence that could lie in data not fully described or interpreted, such as negative or control experiments.

“…none of the tools was able to make a clean distinction between information retrieved from review articles and original research articles.”

## Where do we go from here?

How will scientists have to document the use of these tools? The generation of ideas and hypotheses has not been the focus of scientific documentation or publications. In fact, it is a hallmark of rigorous, high-quality journals to endorse the citation of prior concept illustrations, preprints and the primary literature where appropriate. However, the notion of “ideas are cheap” runs strong in research that relies on costly and laborious experiments—unless a patent is pursued. Often, instrumental suggestions appear as “discussion” in the Acknowledgement section of a typical research article. As such, the advent of LLMs should elicit more discussion and creativity around the documentation of human hypothesis generation. Ever more advanced, LLM-based hypothesis generation tools, also called co-scientists, may have to be documented in supplements to make the instructions and direct output of the system transparent (Penadés et al, 2025).

The literature is the literature with all its glory and its faults, which have resulted in an ongoing discussion on the reproducibility of basic and preclinical research (Begley and Ioannidis, 2015). Nevertheless, efficient and critical literature mining expedites scientific work if our concepts are firm. The section in Peter Medawar’s *Advice to a Young Scientist* on how to choose a meaningful research question is as valid today as it was upon its publication in 1979. The tools discussed here potently support the choice. Using them wisely, scientists will be able to choose their problems effectively. This will save resources, animal lives, and some—but only some—of the dead-ended journeys inherent to science.

The power of the tools must elicit a fundamental discussion on how to define and maintain knowledge of the pertinent fundamental principles required for judgment. Mining the original data reported in the literature, or even better original data available anywhere in a multimodal way, in combination with co-scientists will be magic. AI-based tools using foundation models containing primary data—as much structured and optimized for AI as possible—will lead to much better experiments and will make progress much more efficient. To this end, it would be useful if the number of articles written in natural language were to decrease. Instead, mining existing knowledge should strongly rely on mining databases and structured data figures, whether published as parts of articles or not, rather than abstracts.

“The power of the tools must elicit a fundamental discussion on how to define and maintain knowledge of the pertinent fundamental principles required for judgment.”

The most important conclusion of my interaction with LLM-based tools regards the next generation of scientists. Students should be prepared for the elementary pitfalls, problems and limitations of LLM-based knowledge management and content production. As of now, all outputs of LLM-based tools must be taken with a grain of salt and require cross-validation against each other or classical resources. Curricula in general but also informal training in lab groups have to teach students the basis for extensively using but also understanding the limits of AI-based tools. Developing them further, especially for the analysis of data not yet described in natural language, is an exciting challenge for current and future scientists. This new way of mining the literature is engaging and stimulates creativity. It is here to stay.

## Supplementary information

Peer Review File
