# Supplementary material for: Machines like us scientists? AI tools for mining the scientific literature in basic biomedical science
Source: EMBO Rep. 2025 Jul 10;26(15):3709–13. doi: 10.1038/s44319-025-00522-5 (PMC12331900; doi:10.1038/s44319-025-00522-5)
Supplement: Supplementary file 1 — Peer Review File [file 44319_2025_522_MOESM1_ESM.pdf]

EMBO reports

Machines like us scientists?

Blanche Schwappach

Corresponding author(s): Blanche Schwappach (b.schwappach-pignataro@uke.de)

Review Timeline:

Submission Date:	1st Jul 25
Accepted:	1st Jul 25

Editor: Holger Breithaupt

Transaction Report: This article was editorially reviewed.
